# Essential role of IL-17 in acute exacerbation of pulmonary fibrosis induced by non-typeable *Haemophilus influenzae*

**DOI:** 10.7150/thno.74809

**Published:** 2022-07-04

**Authors:** Shengsen Chen, Xinyun Zhang, Cheng Yang, Shi Wang, Hao Shen

**Affiliations:** 1 Department of Endoscopy (the bronchoscope group), Cancer Hospital of the University of Chinese Academy of Sciences (Zhejiang Cancer Hospital), Institute of Basic Medicine and Cancer (IBMC), Chinese Academy of Sciences, Hangzhou 310022, China.; 2 Department of Microbiology, University of Pennsylvania Perelman School of Medicine, Philadelphia 19104, USA.; 3 Department of Infectious Diseases, Huashan Hospital Affiliated to Fudan University, Shanghai 200040, China.; 4 Department of Infectious Diseases, The First Affiliated Hospital of Chongqing Medical University, Chongqing 400016, China.

**Keywords:** idiopathic pulmonary fibrosis, acute exacerbations, nontypeable* Haemophilus influenzae*, IL-17, γδ T cell

## Abstract

**Background:** Acute exacerbation (AE) of idiopathic pulmonary fibrosis (IPF) has a poor prognosis and lacks effective therapy. Animal models that mimic AE-IPF can greatly accelerate investigation of its pathogenesis and development of effective therapy. However, there are few reports of animal models of AE-IPF caused by bacteria. Thus, our study aimed to establish a mouse model of bacterium‐induced AE‐IPF and explore the potential pathogenic mechanism of AE‐IPF.

**Methods:** Mice were instilled intranasally with bleomycin (BLM) followed by non-typeable* Haemophilus influenzae* (NTHi) strain NT127. Murine survival, bacterial load, body weight and pulmonary histopathological changes were evaluated. We analyzed the T cell and inflammatory cell responses in the lungs.

**Results:** Infection with 10^7^ CFU NT127 triggered AE in mice with PF induced by 30 μg BLM. Compared with BLM-instilled mice, the BLM/NT127-treated mice showed more obvious airway inflammation, lower survival rate, higher inflammatory cell response, and increased proportions and numbers of IL-17^+^CD4^+^, IL-17^+^ γδ T, IL-22^+^CD4^+^ and regulatory T (Treg) cells in lungs. γδ T cells were the predominant source of IL-17. IL-17 gene knockout mice with AE-IPF had quicker body weight recovery, milder pulmonary inflammation and fibrosis, stronger IL-22^+^CD4^+^T, TGF-β^+^ γδ T and Treg cell responses, and weaker neutrophil and eosinophil responses than wild-type mice with AE-IPF.

**Conclusions:** NTHi infection after BLM-induced IPF can cause AE‐IPF in a murine model. This novel model can be used to investigate the pathogenesis of AE‐IPF and develop new therapies for AE‐IPF caused by bacteria. IL-17 is essential for the development of AE-IPF, and it may be a new therapeutic target for bacteria-induced AE-IPF.

## Introduction

Idiopathic pulmonary fibrosis (IPF) is a chronic, progressive and fibrotic interstitial lung disease with unclear etiology 1. IPF patients have poor prognosis, with a median survival time of 2-3 years 2. Acute exacerbation (AE) of IPF is defined as a dramatic decline of respiratory function within 1 month and is the principal (40%) cause of death in patients with IPF 2,3, but its pathogenic mechanism remains unknown 2. Lung histopathology of AE-IPF shows diffuse alveolar damage in addition to fibrosis 1,2. Patients with AE-IPF only have a median survival time of 2-4 months 1,2, and their mortality rate is as high as 50% 2. Specific drugs with sufficient evidence of efficacy are still not available for patients with AE-IPF 4. Therefore, to explore the pathogenesis of AE-IPF is helpful for the development of new, specific therapeutic drugs.

Whether infection, especially bacterial infection, can trigger AE‐IPF is not yet fully elucidated 5. It has been reported that the bacterial load in bronchoalveolar lavage fluid (BALF) of IPF patients is twofold higher than that of healthy controls, which is associated with increased risk for IPF progression 6. Compared with healthy controls, in IPF patients the lower airways are not sterile and are more prone to harbor potentially pathogenic *Haemophilus influenzae*,* Haemophilus parainfluenzae*,* Streptococcus pneumoniae* and *Pseudomonas* 6,7. *H. influenzae* seems to be the common bacterium found in the airway of IPF patients, and it may be related to AE-IPF. Molyneaux et al. found that lung bacterial load in patients with AE-IPF was up to fourfold higher than that in stable IPF patients 8, meaning that alteration of the respiratory bacterial burden may induce the development of AE-IPF. About 89% of bacteria cultured from the sputum of AE-IPF patients are Gram-negative 9.

One study using a mouse model has demonstrated that *S. pneumoniae* (Gram-positive) infection can exacerbate the established IPF 10. Unfortunately, murine models of AE-IPF induced by Gram-negative bacteria (such as *H. influenzae*) have not been established, limiting investigation of the pathogenic mechanism and therapeutic intervention of AE‐IPF triggered by Gram-negative bacteria. Non-typeable* H. Influenzae* (NTHi) is one of the *H. influenzae* strains without encapsulation and commonly colonizes the human respiratory tract 11. NTHi has gradually become the dominant strain because it is not covered by the traditional conjugate vaccines against *H. influenzae* 11. We aimed to establish a novel murine model of AE-IPF induced by NTHi.

In patients with AE-IPF, neutrophils and pro-inflammatory cytokines are substantially increased in BALF 12, and serum levels of pro-inflammatory cytokines are also elevated 13, suggesting that AE-IPF is an inflammatory disease caused primarily by neutrophilic inflammation rather than a fibrotic disease compared to stable IPF. IL-17 is a pro-inflammatory cytokine that induces neutrophilic inflammation 14, which implies that IL-17 participates in the pathogenesis of AE-IPF. Nevertheless, the effect of IL-17 on bacterium-induced AE-IPF has yet to be clarified. We used a novel murine model to further investigate the role of IL-17 in bacterium-induced AE-IPF.

## Methods

### Animals

Specific pathogen-free grade wild-type (WT) C57BL/6 mice were purchased from the National Cancer Institute (Bethesda, MD) and Gempharmatech Co. Ltd. (Nanjing, China). IL-17 gene knockout (KO) C57BL/6 mice were provided by Dr David Artis (Weill Cornell Medical College, New York, NY). Mice were housed in separated cages until age 6-8 weeks and allowed to access water and food freely. The protocols for mouse maintenance and experiments were approved by the Institutional Animal Care and Use Committee in the University of Pennsylvania and Zhejiang Cancer Hospital.

### Bleomycin administration and bacterium infection

Mice were anesthetized by intraperitoneal injections with 100 ml ketamine/xylazine (100 mg/3.8 mg/kg), and administered intranasally with 15, 30 or 60 μg bleomycin (BLM; Acmec Biochemical Co. Ltd., Shanghai, China) in 30 μL phosphate-buffered saline (PBS) to confirm the optimal BLM dose for inducing PF. For lung infection, NTHi strain NT127 was grown in/on brain heart infusion (BHI) broth/agar plates plus 2.0% Fildes Enrichment (BD) and 10 μg/mL NAD (Sigma) at 37 °C. Anesthetized mice were intranasally challenged with 30 μL suspensions of NT127 (10^6^-10^8^ CFU) on day 7 after administration of the optimal BLM dose to establish the model of AE-IPF.

### Clinical observation and histology

Body weight and survival of the mice were recorded daily. The 10 μL serial dilutions of BALF and lung homogenates were plated in triplicate. Twelve hours later, the NT127 CFUs of BALF and lung homogenates were noted on the plates. The harvested lungs were inflated and fixed by intratracheal instillation of 10% formalin. After 24 h of fixation, lung tissue was paraffin-embedded and sectioned for H&E staining, Masson's trichrome staining and immunostaining. Immunostaining was performed using the following antibodies: chicken anti-keratin 5 (Krt5, 1: 200; Covance, Princeton, NJ), rat anti-receptor for advanced glycation end products (RAGE, 1:100; R&D Systems, Minneapolis, MN), rabbit anti-proSurfactant protein C (proSPC, 1:200; Millipore, Billerica, MA), and rabbit anti-αSMA (1:200; Abcam, Cambridge, UK). Slides were mounted with ProLong® Gold mounting medium containing DAPI (Cell Signaling Technology, Danvers, MA).

### Flow cytometry

Lymphocytes from lung or spleen were stained as follows. For surface staining, single-cell suspensions (10^6^-3×10^6^ cells) were stained with anti-mouse antibodies such as anti-CD3, anti-CD4, anti-CD8, anti-γδ TCR, anti-CD25, anti-CD44, anti-CD45, anti-Ly-6C, anti-Ly-6G, anti-CD11b, anti-CD11c, anti-MHC-II, anti-CD24, anti-CD64 and anti-Siglec-F in RPMI 1640 containing 1% fetal bovine serum (FACS buffer) for 30 min at 4 °C, followed with two washes in FACS buffer. After staining of the surface antigens, cells were stained for intracellular/intranuclear cytokines using the BD Cytofix/Cytoperm Kit (BD Pharmingen, San Jose, CA). Antibodies used for intracellular cytokine detection were anti-IL-17, anti-interferon-γ (IFN-γ), anti-transforming growth factor β (TGF-β) and anti-FOXP3. Single-cell suspensions were stimulated *ex vivo* with PMA (50 ng/mL; Sigma-Aldrich, St Louis, MO) and ionomycin (1 μg/mL; Sigma-Aldrich) for 5 h at 37 °C in the presence of BD GolgiStop (BD Biosciences, San Jose, CA). Cells were analyzed using a FACS Canto flow cytometer (BD Biosciences). All antibodies above were purchased from eBioscience (San Diego, CA).

### Statistical analyses

Unpaired, two-tailed, Student's t-tests were used to calculate statistical significance between two groups. The Kaplan-Meier method was used to plot survival curves, and the survival rates were compared by log-rank test. Statistical significance was as follows: *P<0.05; **P<0.01; ***P<0.001; ****P<0.0001. All analyses were performed using GraphPad Prism 8.

## Results

### Murin model of PF development

We identified the optimal dose of BLM to establish the murine model of PF (**Figure S1A**). Mice were intranasally administered BLM at 15, 30 or 60 μg. The body weight of the mice receiving 60 μg BLM dramatically decreased and was hard to recover, whereas the mice administered 15 μg BLM had a slight decrease in body weight and then quickly recovered to normal level (**Figure S1B**). Body weight loss in the 30 μg BLM group was intermediate between that of the other two groups. All mice in the 15 and 30 μg groups survived to the end of follow-up, while the survival rate of the 60 μg group was nearly 20% at the end of follow-up (**Figure S1C**). The histopathological results of lung sections indicated that 60 μg BLM caused lung tissue inflammation on the day 3. The inflammatory lesions extended and were still obvious on day 21 after BLM administration. In the 15 μg BLM group, the inflammatory lesions appeared on day 7, were subsequently relieved, and almost returned to normal on day 21 after BLM administration. The lung inflammatory response induced by 30 μg BLM was milder than that induced by 60 μg BLM but more severe than that induced by 15 μg BLM at the same time point (**Figure S1D**). BLM 30 μg induced an obvious inflammatory response that recovered subsequently, and BLM-instilled mice had a high survival rate with this dose, implying that 30 μg was an optimal dose of BLM for developing the murine model.

Immunofluorescence and Masson's trichrome staining of lung pathological sections were performed to explore the respiratory epithelial cell damage and tissue fibrosis after BLM administration. Destruction of the alveolar epithelial type I (AECI, RAGE^+^) and alveolar epithelial type II (AECII, SPC^+^) cells was observed on days 7 and 3, respectively, after BLM administration, and gradually recovered after 14 days (**Figure S2A and S2B**). Basal cells (Krt5^+^) started to proliferate on day 7, and more basal cells proliferated on day 14 (**Figure S2C**), which may have helped to repair the damage to AECI and AECII cells. Significant fibrotic lesions were observed in lung sections after 30 μg BLM administration, as characterized by myofibroblasts (α-SMA^+^) proliferation and collagen fiber deposition on days 7, 14 and 21 (**Figure S2D and S2E**). These results indicated that 30 μl BLM could definitely induce lung tissue inflammation and fibrosis in a murine model.

### Establishment of AE-IPF murine model

**Figure 1:**
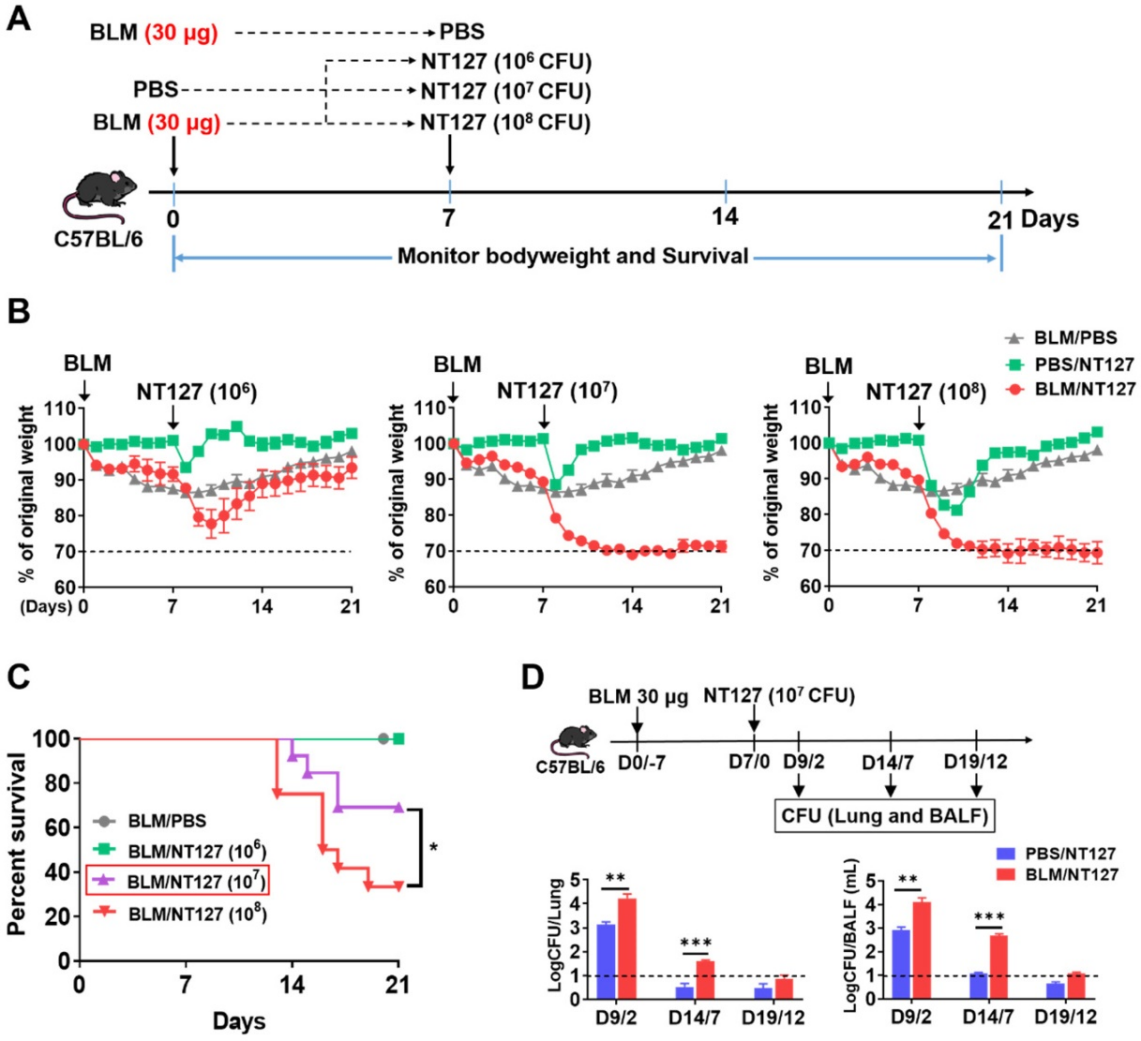
** Murine model of non-typeable* Haemophilus influenzae* NT127-infection-induced acute exacerbation of pulmonary fibrosis.** (A) The workflow of animal modeling. BLM/NT127-treated mice were instilled intranasally with 30 μg BLM. After 7 days, the mice were infected intranasally with 10^6^, 10^7^ or 10^8^ CFU NT127. The BLM-instilled and NT127-infected mice were used as controls. (**B**) Body weight changes in BLM-instilled, NT127-infected and BLM/NT127-treated mice. (**C**) Survival rates comparison among BLM-instilled mice and BLM/NT127-treated mice with 10^6^, 10^7^ and 10^8^ CFU NT127. (**D**) Bacterial loads in murine lung homogenate and bronchoalveolar lavage fluid on 2, 7 and 12 days after NT127 infection. Results are representative of at least three independent experiments with nine or 10 mice in each group. Data shown as mean ± SEM. *P<0.05; **P<0.01; ***P<0.001. BLM: bleomycin.

Immunofluorescence and Masson's trichrome staining in mice receiving 30 μg BLM clearly showed PF on day 7 after administration. The mice were infected with 10^6^, 10^7^ and 10^8^ CFU NT127 at this time point (**Figure 1A**). The body weight of mice administered 30 μg BLM and infected with NT127 10^6^ CFU decreased, to nearly 75% of original weight, then gradually recovered, and returned to about 90% of normal level on day 21. The mice administered 30 μg BLM and infected with 10^7^ or 10^8^ CFU NT127 also showed significant body weight loss, but subsequently had difficult body weight recovery (**Figure 1B**). Mice in the BLM/10^6^ CFU NT127 group all survived at the end of the follow-up. The survival rate of mice in the BLM/10^7^ CFU NT127 group at the endpoint was ~70%, while the survival rate of the BLM /10^8^ CFU NT127 group was <40% (**Figure 1C**). Mice in the BLM/10^7^ CFU NT127 group were unwell for a long time, but had low mortality at the end of follow-up, thus, we chose 30 μg BLM and 10^7^ CFU NT127 to establish the AE-IPF murine model. We investigated the change in bacterial clearance in AE-IPF mice, and found that the bacterial load (lung homogenate and BALF) in the BLM/10^7^ CFU NT127 group was significantly higher than that in the single NT127 infection group, especially on days 2 and 7 after NT127 infection (**Figure 1D**).

### Characteristics of T-cell response in AE-IPF mice

We investigated the T-cell response induced by NTHi infection. The proportion of IL-17^+^ T cells in the lungs increased rapidly after 10^7^ CFU NT127 infection, peaked at day 7, and then decreased (**Figure S3**). The kinetics of the IFN-γ^+^ T-cell response in the lungs were similar to those of IL-17^+^ T cells. However, spleen T cells had a weak response after NT127 infection, and the ratios of IL-17^+^ and IFN-γ^+^ T cells changed at different time points, indicating that the spleen T cells did not play an important role in the specific immune response to NT127 infection. We established the AE-IPF murine model using 30 μg BLM and 10^7^ CFU NT127, killed the mice on day 7 after NT127 infection, and performed flow cytometry to explore the T-cell response in lung tissue (the gating strategy for pulmonary T cells can be seen in **Figure S4**).

**Figure 2:**
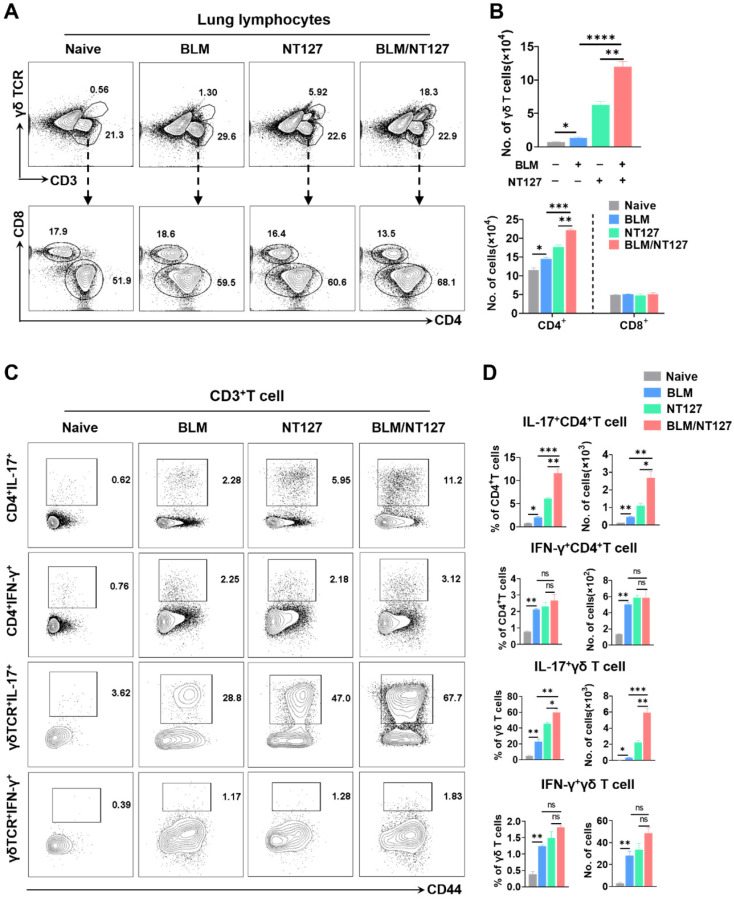
** Characteristics of pulmonary T cells response in mice with acute exacerbation of pulmonary fibrosis.** (**A**) Percentage and (**B**) absolute number of pulmonary CD4^+^, CD8^+^ and γδ T cells in naïve mice, mice treated with BLM alone (BLM), mice infected with non-typeable* Haemophilus influenzae* NT127 alone (NT127), and BLM-treated mice challenged with NT127 (BLM/NT127) on day 7 after NT127 infection (day 14 after BLM administration). IL-17 and IFN-γ production by pulmonary CD4^+^ and γδ T cells after stimulation with PMA/ionomycin as (**C**) visualized by flow cytometry and (**D**) calculated as the percentage and number of IL-17^+^CD4^+^, IFN-γ^+^CD4^+^ , IL-17^+^γδ and IFN-γ^+^ γδ T cells in naïve, BLM, NT127 and BLM/NT127 mice. Data are expressed as the mean±SEM (n = 5 per group), *P<0.05; **P<0.01; ***P<0.001. BLM: bleomycin.

Compared with the BLM-treated mice and NT127-infected mice, those receiving both BLM and NT127 had higher percentages and numbers of CD4^+^ and γδ T cells, but not CD8^+^ T cells in the lungs (**Figure 2A and 2B**). To investigate the function of T cells responding to AE-IPF, cytokine expression was analyzed. IL-17-producing CD4^+^ and γδ T cells obviously expanded in the lungs after challenge with BLM, NT127 and BLM/NT127, and the percentages and numbers of pulmonary IL-17^+^CD4^+^ and IL-17^+^γδ T cells in the BLM/NT127 group were significantly higher than those in the BLM or NT127 groups. In comparison with naïve group, IFN-γ-producing CD4^+^ and γδ T cells expanded in the lungs of the BLM, NT127 and BLM/NT127 groups, but the percentages and numbers among the three groups did not show any significant difference (**Figure 2C and 2D**). In the spleen, low levels of IL-17^+^ and IFN-γ^+^ T cells were detected in the naïve, BLM, NT127 and BLM/NT127 mice. No significant difference was observed in the response levels of splenic IL-17^+^CD3^+^ and IFN-γ^+^CD3^+^ T cells among the BLM, NT127 and BLM/NT127 groups (**Figure S5**). Therefore, AE-IPF induced a strong CD4^+^ and γδ T-cell response that was localized in the lungs and consisted of predominantly IL-17-producing CD4^+^ and γδ T cells mixed with a small population of IFN-γ^+^CD4^+^ and IFN-γ^+^ γδ T cells. There was a minimal IL-22^+^CD4^+^ T-cell and IL-22^+^γδ T-cell response, but the response of IL-22^+^CD4^+^ T cells in BLM/NT127-treated mice was higher (~0.81% CD4^+^ T cells) than that in BLM-treated mice (~0.034% CD4^+^ T cells) or NT127-infected mice (~0.14% CD4^+^ T cells) (**Figure S6**).

### The main source of IL-17 and its critical role in AE-IPF development

**Figure 3:**
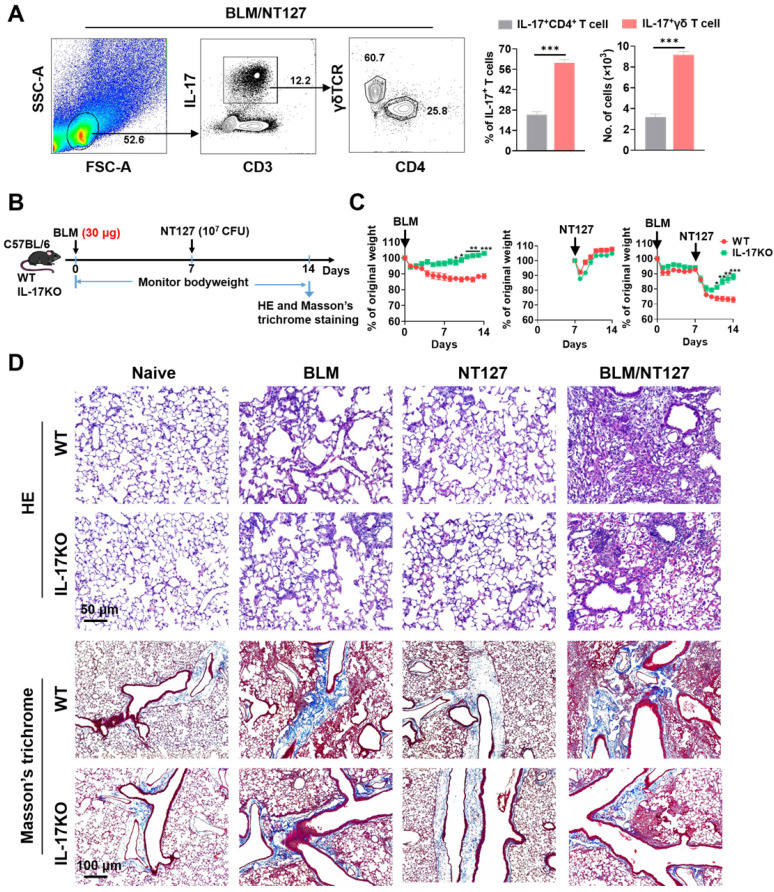
** IL-17 predominantly produced by γδ T cells and its effect on the development of acute exacerbation of pulmonary fibrosis.** (**A**) Percentage and absolute number of pulmonary IL-17^+^ CD4^+^and IL-17^+^γδ T cells in BLM-treated mice infected with non-typeable* Haemophilus influenzae* NT127 were calculated by flow cytometry. (**B**) Schematic of experimental design. (**C**) Body weight changes in WT and IL-17KO mice after intranasally instilling with BLM, NT127 or BLM/NT127. (D) Representative photomicrographs of lung sections stained with H&E and Masson's trichrome on day 7 after NT127 infection (day 14 after BLM instillation) in WT and IL-17KO mice. Scale bars: H&E staining, 50 µm; Masson's trichrome staining, 100 µm. Data are expressed as the mean±SEM (n = 5 per group), *P<0.05; **P<0.01; ***P<0.001. BLM: bleomycin; H&E: hematoxylin and eosin; WT: wild type; KO: knockout.

The above results confirmed that IL-17 came from CD4^+^ and γδ T cells after BLM and NT127 instillation, but it was not clear which type of T cells dominated the secretion of IL-17, so we continued to explore the main source of IL-17 in AE-IPF mice by flow cytometry. IL-17 was mainly secreted by T cells in BLM/NT127-instilled mice, and the percentages and numbers of IL-17^+^γδ T cells were markedly higher than those of IL-17^+^CD4^+^ T cells, suggesting that γδ T cells were the predominant source of IL-17 in AE-IPF (**Figure 3A**). To clarify the role of IL-17 in the development of AE-IPF, we evaluated the changes in body weight and histopathology in IL-17KO mice after BLM and NT127 instillation (**Figure 3B**). The body weight of IL-17KO mice in the BLM and BLM/NT127 groups recovered faster than that of WT mice in the same groups. However, in the NT127-infected group, the body weight recovery of IL-17KO mice was similar to that of WT mice (**Figure 3C**). H&E staining showed that the inflammatory response in lung tissue was mitigated in IL-17KO mice compared with WT mice following instillation of BLM and NT127, yet the fibrotic area was reduced in the IL-17 KO mice with BLM/NT127 instillation according to Masson*'*s trichrome staining (**Figure 3D**). These data indicated that IL‐17 contributed to the NT127‐induced acute inflammation in fibrotic lung.

### IL-22, IFN-γ and TGF-β expression from CD4+ and γδ T cells in IL-17-deficient AE-IPF mice

**Figure 4:**
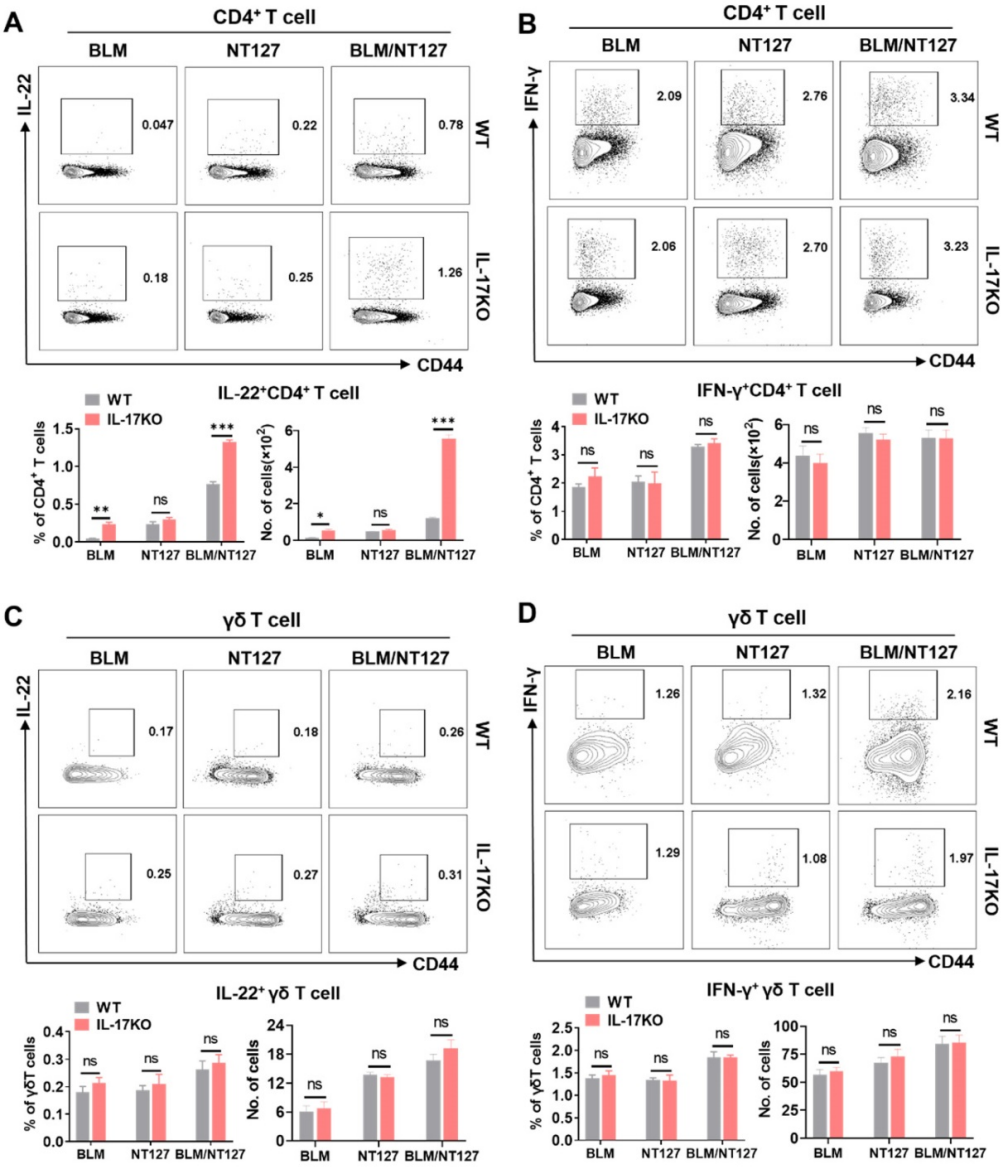
** Impact of IL-17 on IL-22 and IFN-γ production by pulmonary CD4^+^ and γδ T cells in mice with acute exacerbation of pulmonary fibrosis.** IL-22 and IFN-γ expression by pulmonary CD4^+^ and γδ T cells after stimulation with PMA/ionomycin in WT and IL-17 KO mice were analyzed by flow cytometry and calculated as the percentage and number of IL-22^+^CD4^+^ (**A**), IFN-γ^+^CD4^+^ (**B**), IL-22^+^ γδ (**C**) and IFN-γ^+^ γδ T cells (**D**) after intranasally instilling with BLM, NT127 and BLM/NT127. Data are expressed as the mean ± SEM (n = 5 per group), *P<0.05; **P<0.01; ***P<0.001. BLM: bleomycin; WT: wild type; KO: knockout.

Secretion of IL-17, IFN-γ and IL-22 from pulmonary CD4^+^ and γδ T cells in WT mice after BLM and NT127 instillation is shown in **Figure 2 and Figure S6**. Whether deficiency of IL-17 expression influenced the levels of IL-22 and IFN-γ produced from pulmonary CD4^+^ and γδ T cells in AE-IPF mice remained unclear. Thus, we assessed the responses of IL-22^+^CD4^+^, IFN-γ^+^CD4^+^, IL-22^+^ γδ and IFN-γ^+^ γδ T cells in the lungs of IL-17KO mice to identify whether the loss of IL-17 expression affected secretion of IL-22 and IFN-γ following BLM/NT127 instillation. Flow cytometry showed that the percentages and numbers of IL-22^+^CD4^+^ T cells tended to be higher in IL-17KO mice than those in WT mice after BLM or BLM/NT127 instillation (**Figure 4A**). Nevertheless, the differences in responses of IFN-γ^+^CD4^+^, IL-22^+^γδ^+^ and IFN-γ^+^ γδ T cells in lungs between WT and IL-17KO mice were not significant following BLM, NT127 and BLM/NT127 instillation (**Figure 4B-D**). These data implied that IL-17 might inhibit expression of IL-22 from CD4^+^ T cells but not γδ T cells in AE-IPF mice, and secretion of IFN-γ from CD4^+^ and γδ T cells was not influenced by IL-17 after BLM/NT127 instillation. Given that TGF-β is strongly related to IL-17 and IL-22 expression 15, we investigated TGF-β production in IL-17KO mice with AE-IPF. The percentages and numbers of TGF-β^+^γδ T cells rather than TGF-β^+^CD4^+^ T cells were significantly higher in IL-17KO mice than those in WT mice after BLM or BLM/NT127 instillation (**Figure S7**).

### Effects of IL-17 on response of inflammatory and Treg cells in AE-IPF mice

**Figure 5:**
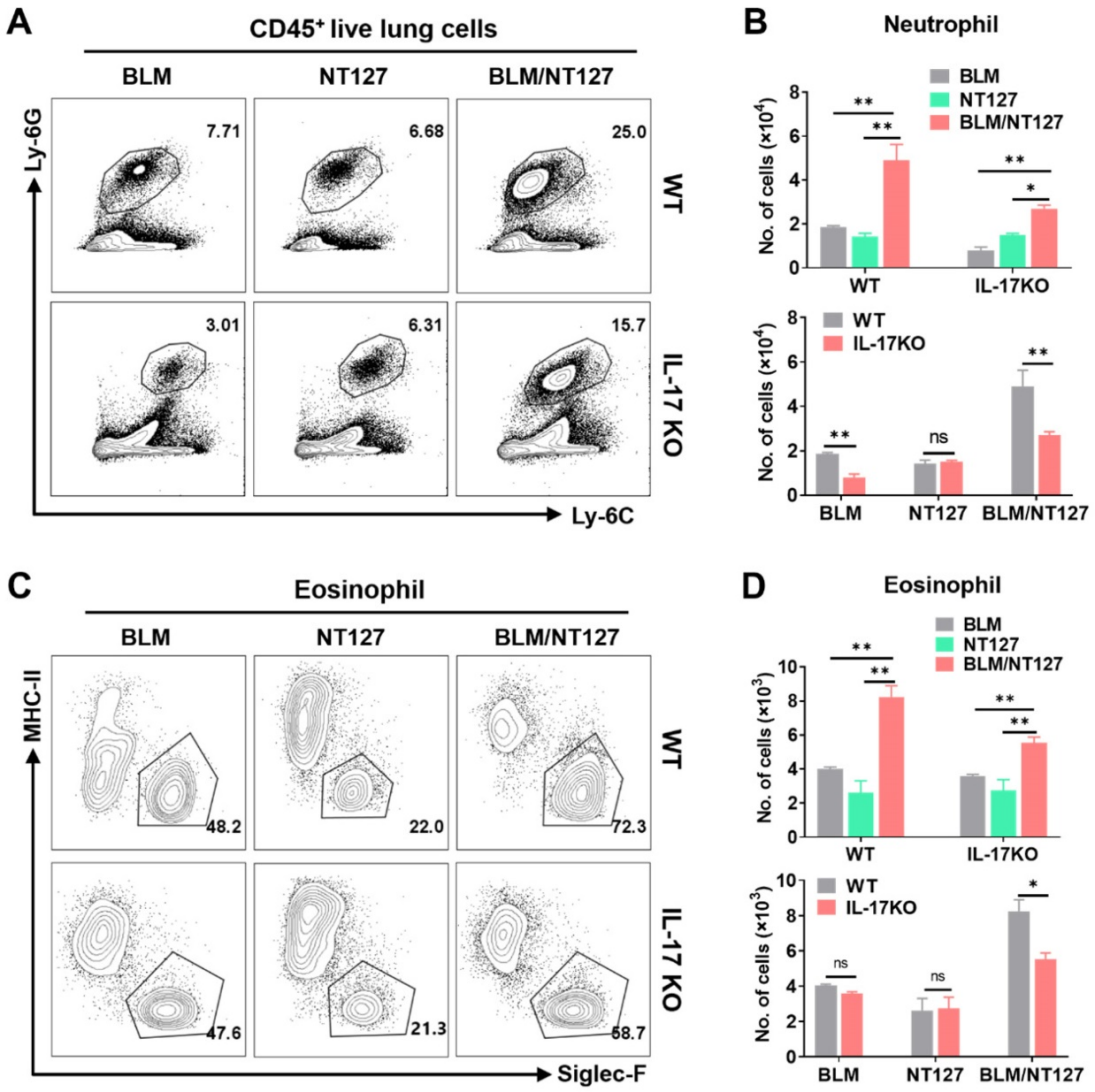
** Effects of IL-17 on the responses of neutrophils and eosinophils in the lungs of mice with acute exacerbation of pulmonary fibrosis.** Absolute number of pulmonary neutrophils (**A** and **B**) and eosinophils (**C** and **D**) was calculated by flow cytometry in WT and IL-17 KO mice after intranasally instilling with BLM alone, NT127 alone, and BLM/NT127. Data are expressed as the mean ± SEM (n = 5 per group), *P<0.05; **P<0.01; ***P<0.001. BLM: bleomycin; WT: wild type; KO: knockout.

**Figure 6:**
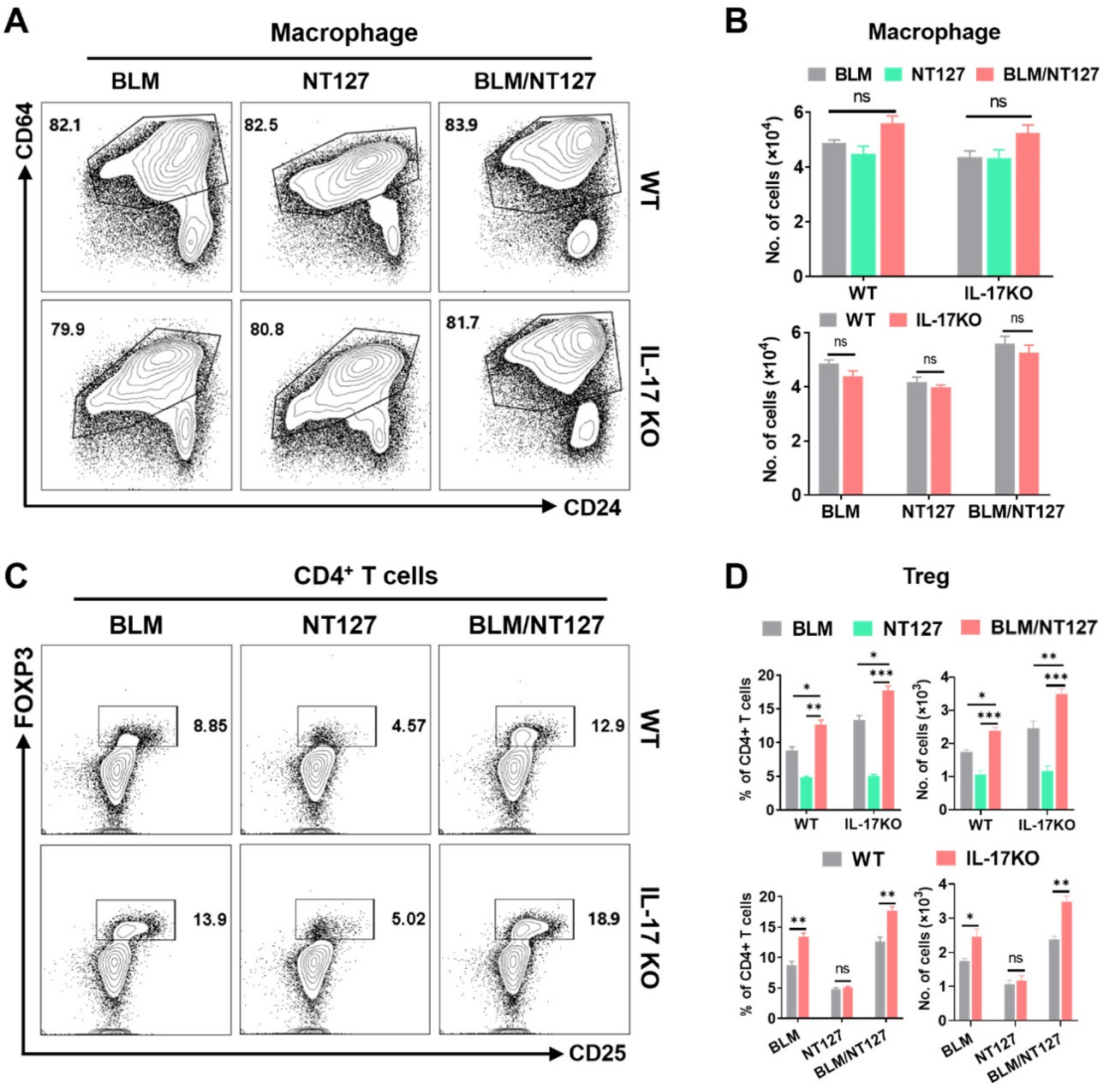
**Effects of IL-17 on the response of macrophages and Treg cells in the lungs of mice with acute exacerbation of pulmonary fibrosis.** In WT and IL-17 KO mice after intranasally instilling with BLM alone, NT127 alone, and BLM/NT127, the absolute number of pulmonary macrophages (**A** and **B**), and the percentage and absolute number of pulmonary Treg cells (**C** and **D**) were analyzed by flow cytometry. Data are expressed as the mean ± SEM (n = 5 per group), *P<0.05; **P<0.01; ***P<0.001. BLM: bleomycin; Treg cells: T regulatory cells; WT: wild type; KO: knockout.

IL-17 plays an important role in the pulmonary inflammation and fibrosis caused by BLM and NT127 (**Figure 3C and 3D**), but the pro-inflammatory mechanism of IL-17 in AE-IPF mice is still unknown. We investigated the effect of IL-17 on the responses of major inflammatory cells (neutrophils, eosinophils and macrophages) using flow cytometry (the gating strategy for pulmonary inflammatory cells is shown in **Figure S8**). The numbers of neutrophils and eosinophils in the lungs were substantially increased in the BLM/NT127-treated mice compared with BLM-treated or NT127-infected mice, indicating that recruitment of inflammatory neutrophils and eosinophils in the lungs may be associated with the severity of lung damage. Furthermore, in the BLM/NT127 group, IL-17KO mice had less accumulation of neutrophils and eosinophils in the lungs than WT mice had (**Figure 5**). However, macrophage response in the lungs of the BLM/NT127 group was not significantly different from that in the BLM and NT127 groups, and in the three groups, the number of macrophages in IL-17KO mice was similar to that in WT mice (**Figure 6A and 6B**).

It is well known that Treg cells have anti-inflammatory effects and can inhibit excessive immune responses 16. Therefore, we finally detected the responses of Treg cells in AE-IPF mice, and explored whether Treg cells were influenced by IL-17 after BLM/NT127 instillation. Flow cytometry showed that the percentages and numbers of Treg cells in the BLM/NT127 group were higher than those in the BLM and NT127 groups, suggesting that the severe inflammatory response in the BLM/NT127 group promoted the proliferation and accumulation of Treg cells to suppress pulmonary inflammation. In BLM and BLM/NT127 groups, the response of Treg cells in IL-17KO mice was significantly stronger than that in WT mice, which suggested that IL-17 had an inhibitory effect on Treg cells, while the IL-17KO and WT mice in the NT127 group showed similar Treg cell responses (**Figure 6C and 6D**). Collectively, these results suggest that BLM/NT127-induced AE-IPF is associated with IL-17-promoted neutrophil and eosinophil chemotaxis towards the lungs and IL-17-suppressed Treg-cell response in the lungs.

## Discussion

AE-IPF is categorized as triggered AE and idiopathic AE; triggered AE can be sparked by infection (bacteria and viruses), surgery, drugs and aspiration 2,17. Evidence of infection by Gram-negative bacteria (~89% of total bacteria) such as *H. influenzae* has been found in the airways of AE-IPF patients 5,9. The prognosis of AE-IPF is poor 18, with an elusive etiology and pathogenesis, and no effective therapy and specific drugs until now 4,19. Animal models that accurately mimic AE-IPF can facilitate the investigation of AE-IPF pathogenesis and development of effective therapeutic strategies. Although the animal models of AE-IPF induced by non-infection, and viral and Gram-positive bacterial infection have been reported previously 10,20,21, Gram-negative bacteria-induced AE-IPF animal models are still absent, which seriously restricts research into pathogenesis and effective therapy. Therefore, in this study, we sought to establish a murine model of AE-IPF induced by NTHi in addition to BLM administration. To our knowledge, this is the first study to establish a model of AE-IPF induced by a Gram-negative bacterium NTHi, and to explore the role of NTHi in AE-IPF.

Using this novel murine model of AE-IPF, we found that body weight and survival rate of mice infected with NT127 after BLM instillation significantly decreased compared with that of the mice with BLM instillation alone. The bacterial burden in the lung tissue and BALF of mice instilled with BLM/NT127 was higher than that of mice infected with NT127 alone, which was consistent with previous reports that IPF patients have an increased bacterial burden in BALF, predicting pulmonary functional decline and death 6,22, suggesting that the bacterial burden is associated with AE-IPF. BLM/NT127-instilled mice had more inflammatory cell infiltration in the lung tissue and more severe airway structural damage than BLM-treated mice and NT127-infected mice. Therefore, NT127-induced exacerbation of stable PF caused by BLM resembled the clinical presentations of patients with AE‐IPF, indicating that the AE-IPF murine model constructed by BLM/NT127 instillation mimics AE‐IPF development in human patients.

Subsequently, we used this murine model to investigate the potential immune mechanism of AE-IPF induced by NTHi infection, and found that CD4^+^ and γδ T cells strongly responded in the lungs, but not the spleens of mice with AE-IPF. We further explored the function of CD4^+^ and γδ T cell responses in AE-IPF, and our data showed that the levels of IL-17 secreted from CD4^+^T and γδ T cells were significantly increased after NT127 infection following BLM instillation. Unexpectedly, γδ T cells were confirmed as the predominant cell secreting IL-17. The secretion levels of IFN-γ from pulmonary CD4^+^ and γδ T cells were increased in the BLM, NT127 and BLM/NT127 groups compared with the naïve group, but the percentages and numbers of IFN-γ^+^CD4^+^ and IFN-γ^+^ γδ T cells did not differ significantly among the BLM, NT127 and BLM/NT127 groups. This indicated that the secretion of IFN-γ in pulmonary CD4^+^ and γδ T cells might be sparked by non-specific inflammation. The BLM, NT127 and BLM/NT127 groups only had weak IL-22^+^CD4^+^ and IL-22^+^ γδ T cells responses in the lungs, but the response level of pulmonary IL-22^+^CD4^+^ T cells in the BLM/NT127 group was higher than that in the BLM and NT127 groups.

The role of IL-17 in the occurrence and development of respiratory diseases has attracted widespread attention 23. IL-17 has been identified as a proinflammatory factor, and it is closely correlated with acute pulmonary inflammation 14,24. Our results showed that the body weight of WT mice was more difficult to recover than that of IL-17KO mice after BLM/NT127 instillation. Compared with WT mice with AE-IPF, H&E staining revealed that lung tissue inflammatory response in IL-17KO mice was attenuated surprisingly, and Masson's trichrome staining showed that collagen deposition in the lungs of IL-17KO mice with AE-IPF was reduced. This suggests that IL-17 plays a critical role in AE-IPF development after BLM/NT127 instillation. In the BLM and BLM/NT127 groups, the level of IL-22 secreted by pulmonary CD4^+^ T cells in IL-17KO mice was significantly higher than that of WT mice, despite the weak response of pulmonary IL-22^+^CD4^+^ T cells. Sonnenberg et al. identifed that IL-22 had a pro-inflammatory effect on the airway damage with presence of IL-17, but the absence of IL-17 enhanced IL-22 expression and caused it to play a tissue-protective role in airway inflammation 25. Collectively, production of IL-22 from pulmonary CD4^+^ T cells is affected by IL-17 in BLM/NT127-induced AE-IPF, and the increased expression of IL-22 in the absence of IL-17 may have contributed to the protection against airway damage, but the definitive role of IL-22 in AE-IPF remains to be determined. In contrast, in the BLM, NT127 and BLM/NT127 groups, the levels of IFN-γ secreted from pulmonary CD4^+^ and γδ T cells of IL-17KO mice were not significantly different from those of WT mice, suggesting that IL-17 did not influence the production of IFN-γ from CD4^+^ and γδ T cells in the lungs of BLM/NT127-instilled mice.

As for the impact of TGF-β on IL-22 production by T cells, the existing reports are contradictory. Previous studies have identified that TGF-β can promote T-cell-derived IL-22 expression *in vitro* 26-28. Other publications have shown that TGF-β might suppress IL-22 production *in vitro* 29-31. In this study, we found that the percentages and numbers of TGF-β^+^ γδ T cells were significantly higher in IL-17KO mice than in WT mice after BLM or BLM/NT127 instillation. TGF-β may have played a role in promoting the expression of IL-22 in our AE-IPF model, and led to the increase of CD4^+^T cell-derived IL-22. TGF-β can also inhibit inflammation 32. The strong inflammatory response in the lungs of AE-IPF mice may have caused TGF-β expression in a feedback manner. In addition, TGF-β is an important factor required for IL-17 expression 15. In IL-17-deficient AE-IPF mice, IL-17 depletion may induce TGF-β production in a feedback manner. Therefore, under the dual feedback effects of inflammatory environment and IL-17 deficiency, the expression of TGF-β in IL-17KO mice with AE-IPF is significantly increased. Unfortunately, this hypothesis and the specific molecular mechanism about the interaction of IL-17, IL-22 and TGF-β still needs to be further explored.

**Figure 7:**
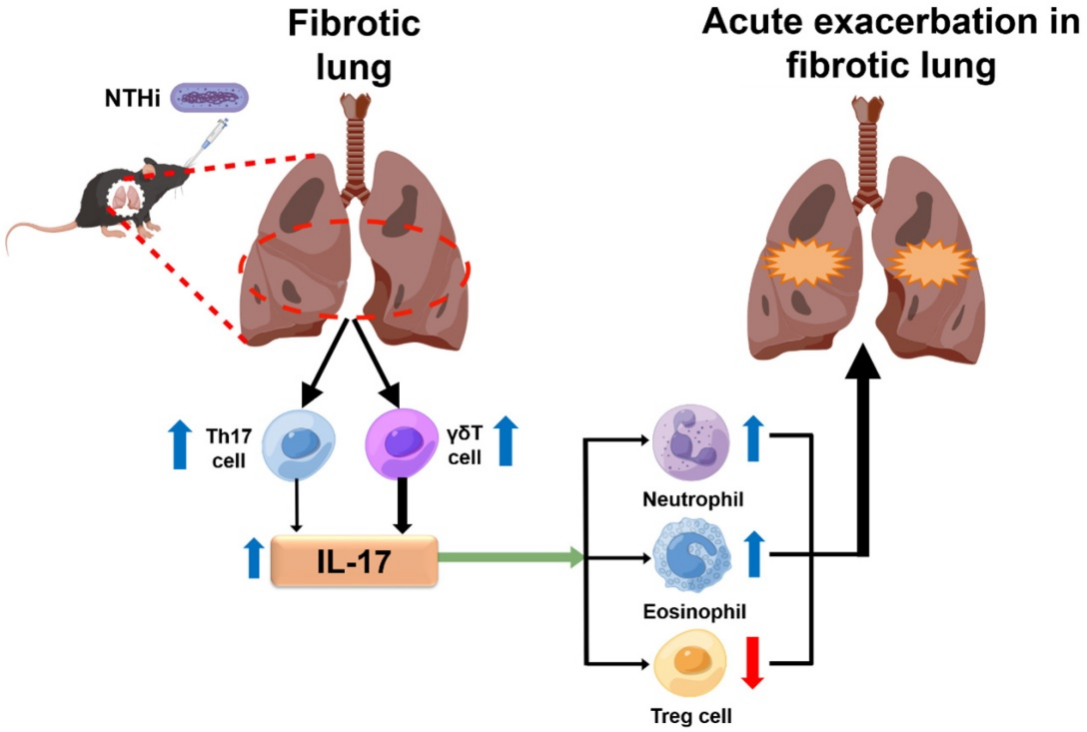
** Schematic illustration of the potential mechanism highlighting the role of IL-17 in NTHi-induced acute exacerbation of pulmonary fibrosis.** NTHi infection can induce a large amount of IL-17 secretion in the fibrotic lung, which predominantly produced from γδ T cells, and a small part of IL-17 secreted from Th17 cells. The high level of IL-17 may promote pulmonary inflammatory damage by recruiting neutrophils and eosinophils to the lung and inhibiting the response of pulmonary T regulatory cells. The blue arrow indicates a high level and the red arrow means a low level. NTHi: non-typeable *H. influenzae*. This figure is originated from Figdraw (www.figdraw.com) with permission ID RPWPTda4bd.

IL-17 has been reported to promote the activation of inflammatory cells like neutrophils, eosinophils and macrophages to exacerbate airway inflammation 33-35. Responses of inflammatory cells also have an effect on pathogen clearance 23,36. Hence, inflammatory cells are involved in maintaining the balance between pathogen elimination and aggravation of acute pulmonary inflammation in an IL‐17‐dependent manner. We speculated that, if the balance shifted to aggravate acute lung inflammation, IL-17 may not act in pathogen clearance. Our study revealed that IL-17 level and bacterial burden (lung homogenate and BALF) in BLM/NT127 mice were both higher than those in NT127 mice, which supported this speculation. Treg cells play a key role in suppressing immune inflammatory responses 16, and many clinical studies or animal experiments have confirmed IL-17 elevation accompanied by Treg cell reduction in autoimmune diseases 37,38, implying that IL-17 may promote inflammation and exacerbate tissue damage by inhibiting Treg cells. Therefore, we explored the influence of IL-17 on infiltration of neutrophils, eosinophils, macrophages and Treg cells in lungs after BLM/NTHi instillation. The number of pulmonary neutrophils and eosinophils in the BLM/NT127 group was significantly higher than that in the BLM and NT127 groups, while no significant difference was observed in macrophages among the three groups. Fewer neutrophils and eosinophils infiltrated the lungs of IL-17KO mice than WT mice with BLM/NT127 instillation, whereas the number of pulmonary macrophages did not significantly differ between IL-17KO mice and WT mice in the three groups. It needs to be emphasized that neutrophils, eosinophils and macrophages do not comprise all inflammatory cells in the lungs. Whether other inflammatory cells contribute to the BLM/NT127-induced pulmonary inflammation remains to be elucidated. A stronger response of Treg cells was found in the lungs of IL-17KO mice than in WT mice after instillation of BLM/NT127. TGF-β is essential for Treg cell differentiation 39. In our AE-IPF model, IL-17KO mice had a higher level of TGF-β than WT mice had. The enhanced response of Treg cells may be associated with the high level TGF-β in IL-17KO mice with AE-IPF. However, the mechanism of how IL-17 regulates Treg cell response by affecting TGF-β production is still unclear. Taken together, our results suggest that IL‐17 produced from Th17 (IL-17^+^CD4^+^) and γδ T cells could be the pivotal mediator for the development of BLM/NT127-infection-induced AE-IPF, and it promotes pulmonary inflammatory damage by recruiting neutrophils and eosinophils to the lung and inhibiting the response of pulmonary Treg cells (**Figure 7**). Nevertheless, the response of pulmonary macrophages was not affected by IL-17 in BLM/NT127-induced AE-IPF. It is disappointing that how IL-17 regulates inflammatory cells (neutrophils and eosinophils) to aggravate lung tissue inflammation and induce PF in our model is still unanswered. More importantly, the detailed roles and mechanisms of Treg cells, neutrophils and eosinophils in the pathogenesis of AE-IPF in BLM/NT127-treated mice remain unknown and need to be further investigated.

In summary, the main innovation of our study was that a murine model of AE-IPF induced by a Gram-negative bacterium was established by infection with NTHi strain NT127 following BLM administration. The model mimics human AE‐IPF and could be an excellent tool to investigate potential mechanisms of host-bacteria interactions, providing the first causal evidence that bacteria, especially Gram-negative bacteria, participate in AE-IPF. We demonstrated that IL-17 played an essential role in AE-IPF by using this murine model. BLM/NT127 instillation induced a large amount of IL-17 secretion, most of which was produced from γδ T cells, and the remainder was derived from CD4^+^ T cells. Loss of IL-17 ameliorated airway inflammation and PF in this model by reducing the number of pulmonary neutrophils and eosinophils, and enhancing Treg cell response in the lungs, which suggests that IL-17 is a potential therapeutic target for AE-IPF induced by NTHi.

## Supplementary Material

Supplementary figures.

## Abbreviations

AE: acute exacerbation
IPF: idiopathic pulmonary fibrosis
BLM: bleomycin
NTHi: non-typeable Haemophilus influenzae
BALF: bronchoalveolar lavage fluid
WT: wild‐type
KO: knockout
BHI: brain heart infusion
CFU: colony-forming unit
H&E: hematoxylin & eosin
TGF-β: Transforming growth factor β
