# Supplementary material for: Essential role of IL-17 in acute exacerbation of pulmonary fibrosis induced by non-typeable Haemophilus influenzae
Source: Theranostics. 2022 Jul 4;12(11):5125–37. doi: 10.7150/thno.74809 (PMC9274745; doi:10.7150/thno.74809)
Supplement: Supplementary file 1 — Supplementary figures. [file thnov12p5125s1.pdf]

Essential role of IL-17 in acute exacerbation of pulmonary fibrosis induced by non-typeable *Haemophilus influenzae*

Shengsen Chen[1,2†], Xinyun Zhang[2,3†], Cheng Yang[2,4], Shi Wang[1*], Hao Shen[2*]

[1]Department of Endoscopy (the bronchoscope group), Cancer Hospital of the University of Chinese Academy of Sciences (Zhejiang Cancer Hospital), Institute of Basic Medicine and Cancer (IBMC), Chinese Academy of Sciences, Hangzhou 310022, China.
[2]Department of Microbiology, University of Pennsylvania Perelman School of Medicine, Philadelphia 19104, USA.
[3]Department of Infectious Diseases, Huashan Hospital Affiliated to Fudan University, Shanghai 200040, China.
[4]Department of Infectious Diseases, The First Affiliated Hospital of Chongqing Medical University, Chongqing 400016, China.

[*]Corresponding author:
Shi Wang, Department of Endoscopy (the bronchoscope group), Zhejiang Cancer Hospital, No. 1 Banshandong Road, Hangzhou 310022, China. E-mail: wangshi@zjcc.org.cn;
Hao Shen, Department of Microbiology, University of Pennsylvania Perelman School of Medicine, 3610 Hamilton Walk, Philadelphia 19104, USA. E-mail: hshen@mail.med.upenn.edu

[†]These authors are contributed equally to this work.

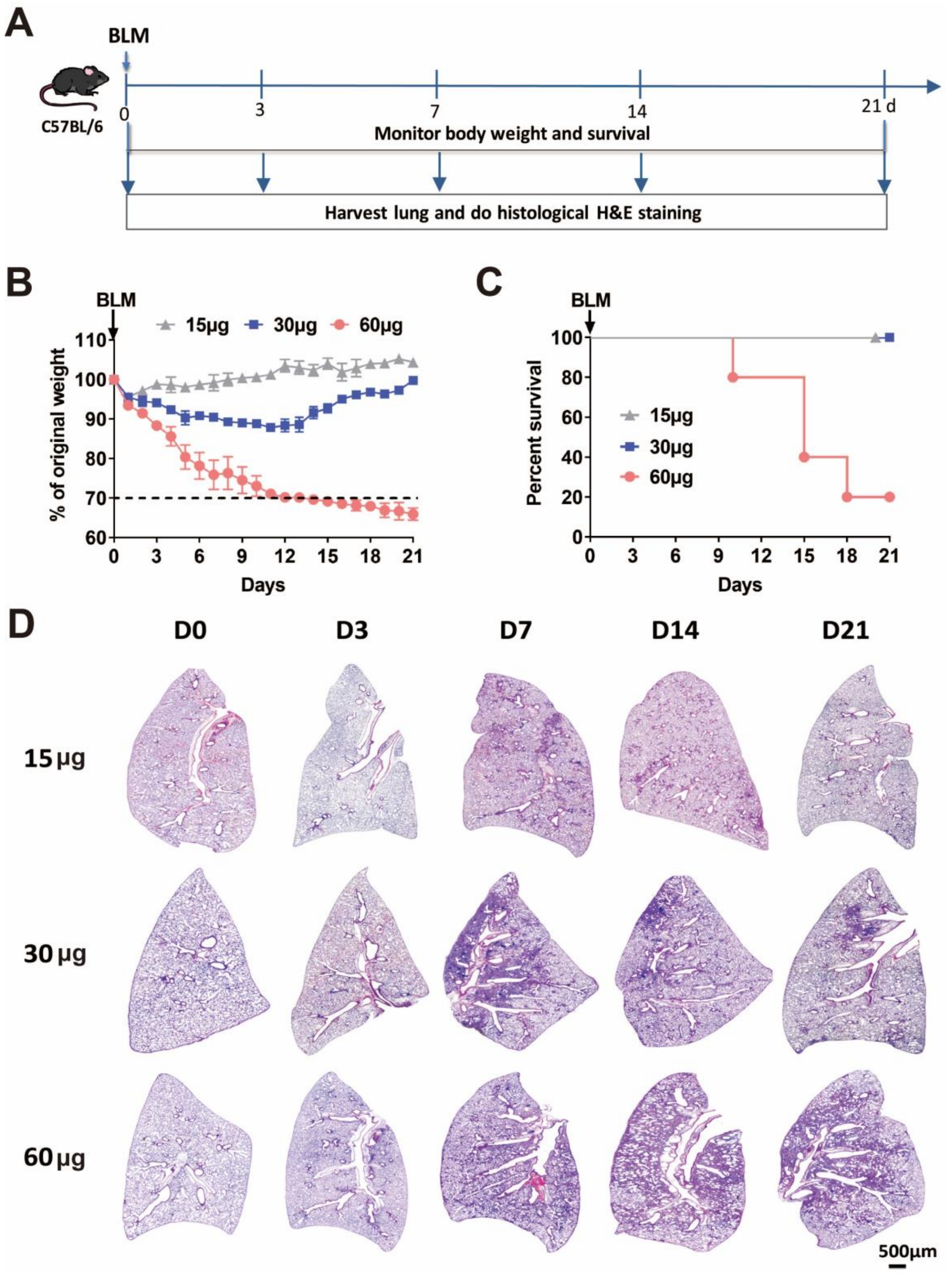

Figure S1. Establishment of pulmonary fibrosis mouse model. (A) The diagram illustrating the establishment of the mouse model of bleomycin (BLM)-induced pulmonary fibrosis. **(B)** Body weight changes of the mice intranasally administrated with BLM in dosages of 60 μg, 30 μg and 15 μg per mouse severally. **(C)** Survival rate comparison among the mice intranasally administrated with BLM in dosage of 60 μg, 30 μg and 15 μg per mouse severally. **(D)** Representative images of lung sections stained with H&E on day 0, 3, 7, 14 and 21 after BLM administration in the dosages of 15, 30 and 60 μg per mouse severally (scale bar: 500 μm). Data are expressed as the mean±SEM of 5-10 mice/group. *P < 0.05; **P < 0.01; ***P < 0.001.

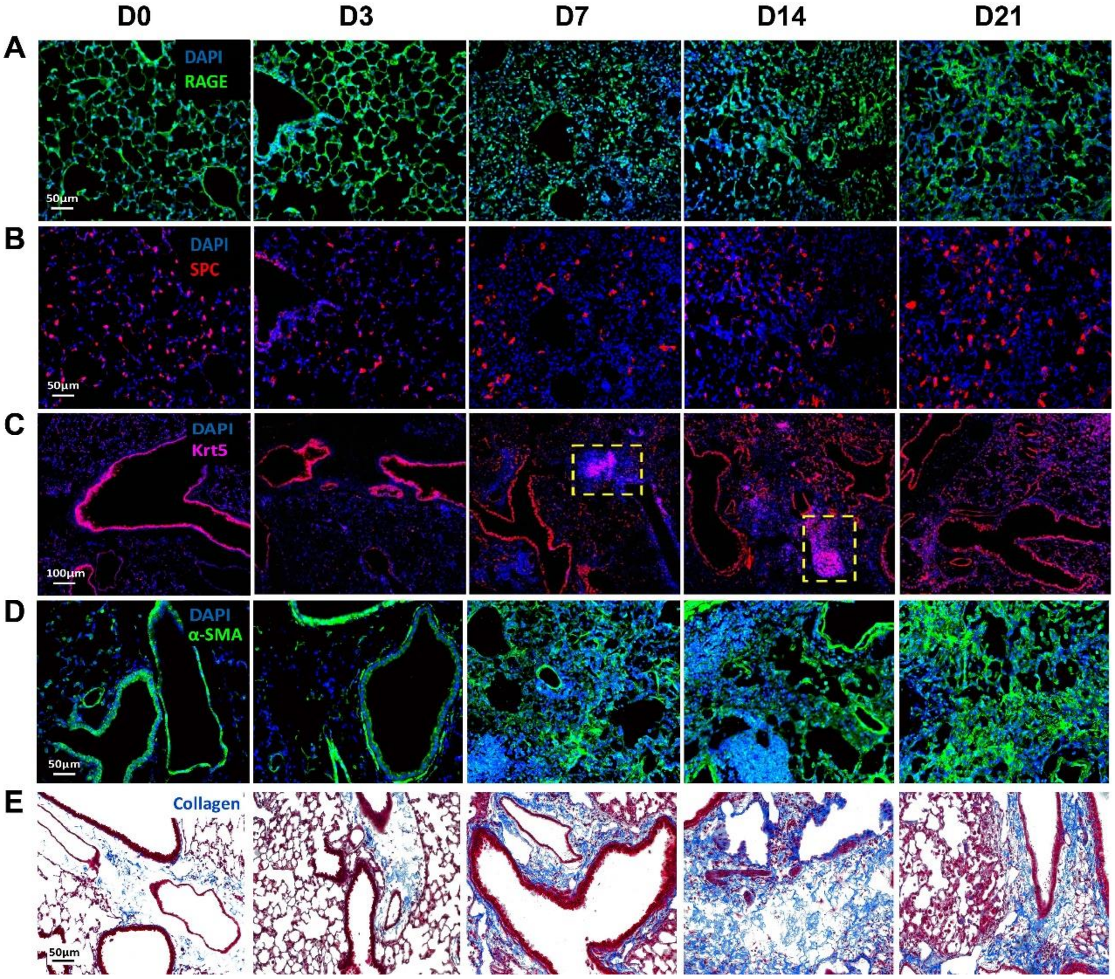

Figure S2. The airway damage and fibrosis induced by instillation of BLM. Lung tissues were collected on day 0, 3, 7, 14, and 21 after 30 µg BLM administration. **(A and B)** Immunostaining with an antibody to the alveolar epithelial type I cells (AECI, $RAGE^{+}$) and alveolar epithelial type II cells (AECII, SPC^{+}). **(C)** Histological analysis of lung sections showing proliferating basal cells ($Krt5^{+}$, area within the yellow dotted line) on day 7 and 14 after BLM administration. **(D and E)** Pulmonary fibrosis was evaluated by the immunostaining with an antibody to myofibroblasts (α-SMA^{+}) and Masson's trichrome staining specific for collagen fibers. DAPI: represents the nucleus. Scale bars: A, B, D and E: 50 µm; C: 100 µm.

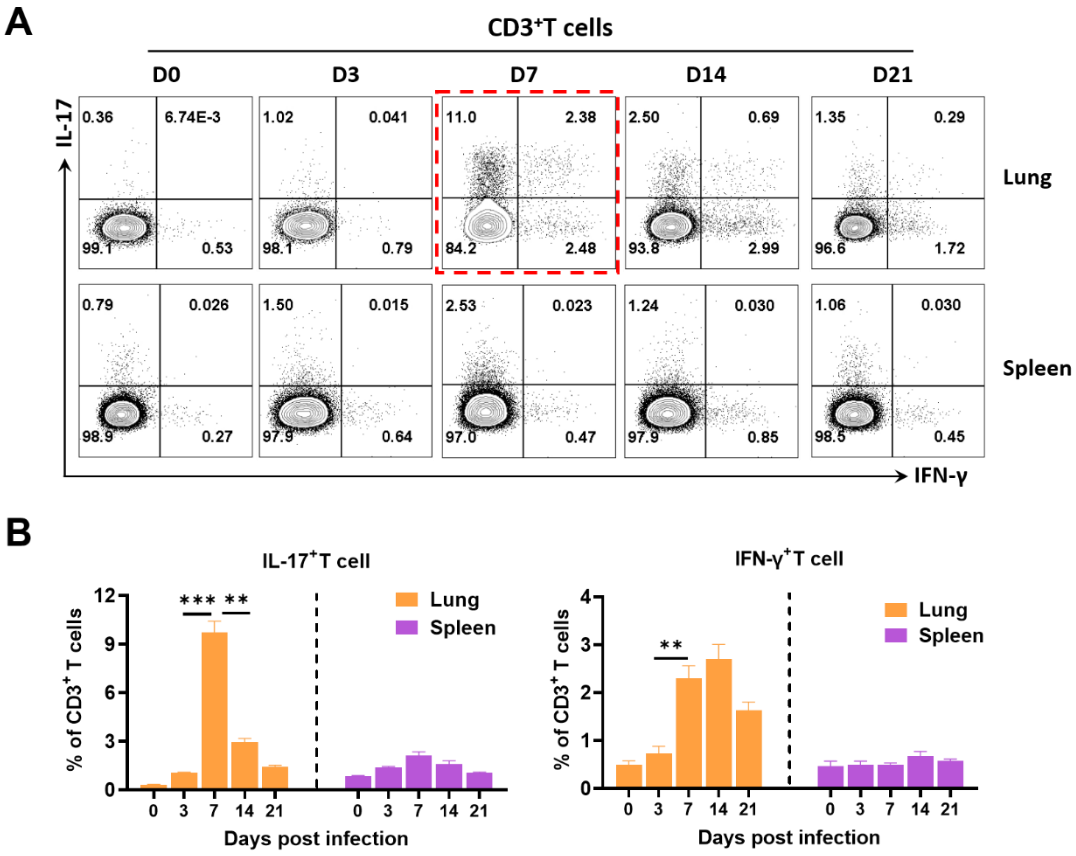

Figure S3. Kinetics of T cell response to NT127 infection. (A) Kinetics of NT127-specific T cells producing IL-17 (IL-17^{+}T cell) and IFN-γ (IFN-γ^{+}T cell) were determined in the lung and spleen of mice. **(B)** Statistical analyses for the results of (A). Data are expressed as the mean±SEM (n=5 mice/group). *P < 0.05; **P < 0.01; ***P < 0.001.

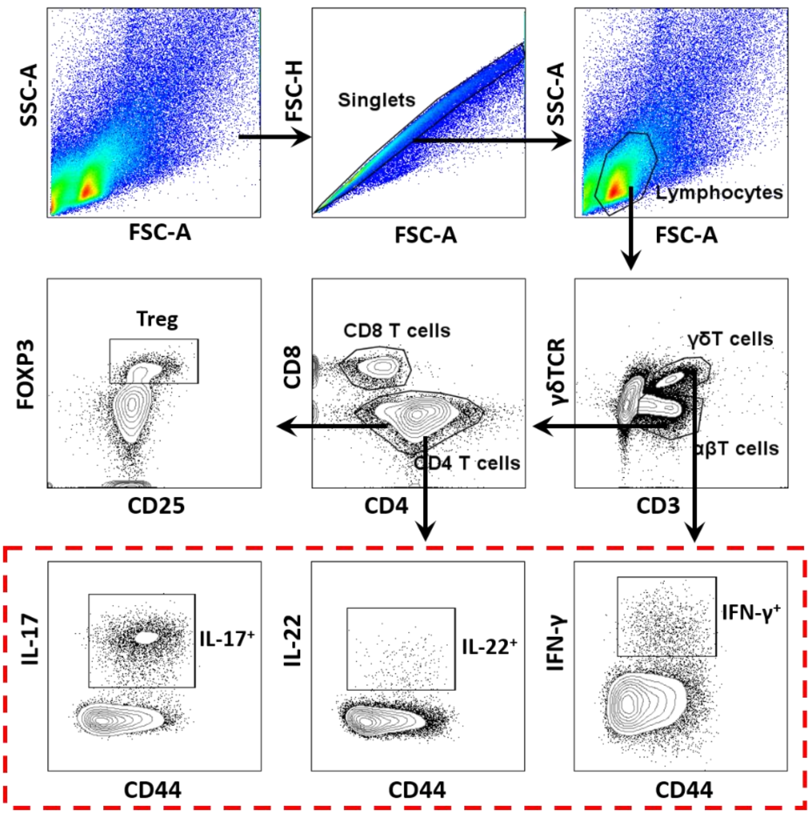

Figure S4. Gating strategy used for the identification of cytokines-producing T cell subsets in murine lung.

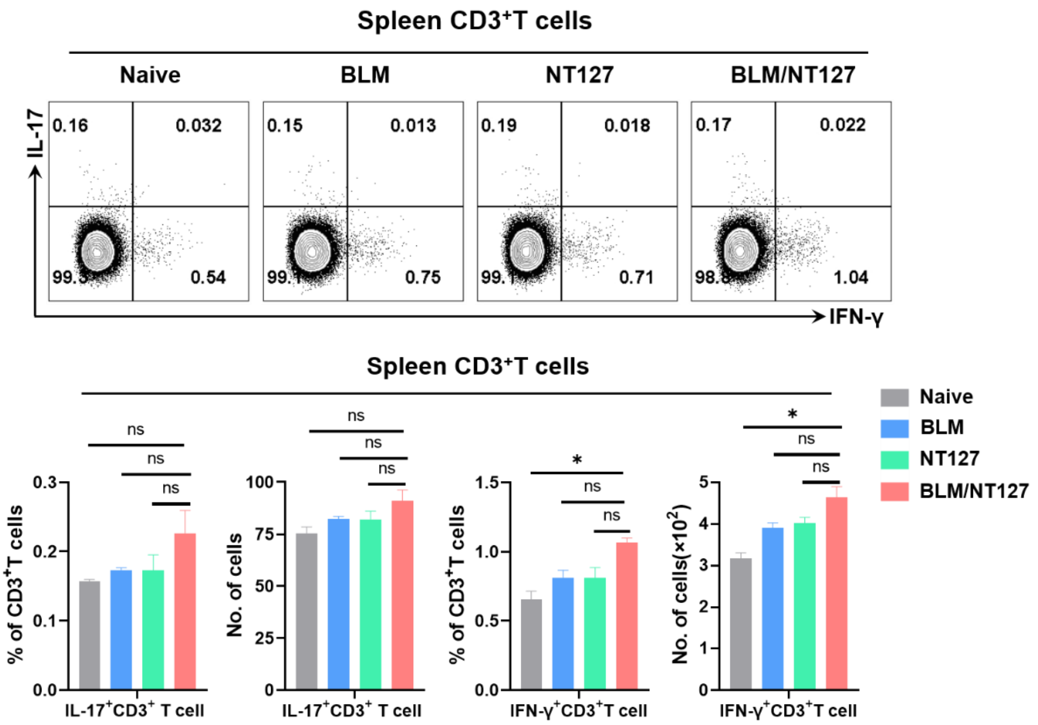

Figure S5. The characteristics of splenic T cells response in AE-IPF mice. IL-17 and IFN-γ production by total T cells ($CD3^+$) after stimulation with PMA/Ionomcyin as visualized by flow cytometry and calculated as the percentage and number of IL-17^+CD3^+ and IFN-γ^+CD3^+ in the speen of naive, BLM-instilled, NT127-infected and BLM/NT127-instilled mice on day 7 after NT127 infection (day 14 after BLM administration). Data are expressed as the mean±SEM(n=5 per group), *P < 0.05; **P < 0.01; ***P < 0.001.

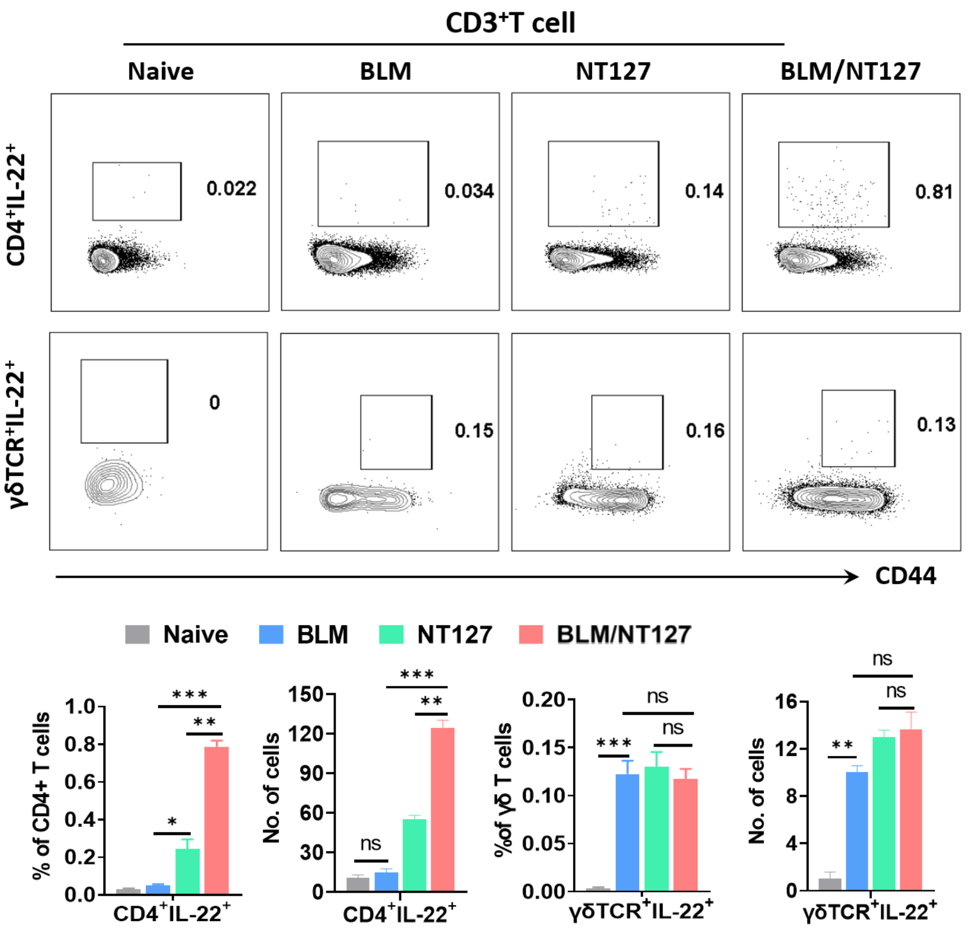

Figure S6. Weak IL-22-producing CD4$^+$ and γδ T cells response in the lung. IL-22 secretion by pulmonary CD4$^+$ and γδT cells after stimulation with PMA/Ionomcyin as visualized by flow cytometry and calculated as the percentage and number of IL-17$^+$CD4$^+$, IFN-γ$^+$CD4$^+$, IL-17$^+$γδ in naive, BLM, NT127 and BLM/NT127 mice on day 7 after NT127 infection (day 14 after BLM administration). Data are expressed as the mean ±SEM (n=5 per group), *P < 0.05; **P < 0.01; ***P < 0.001.

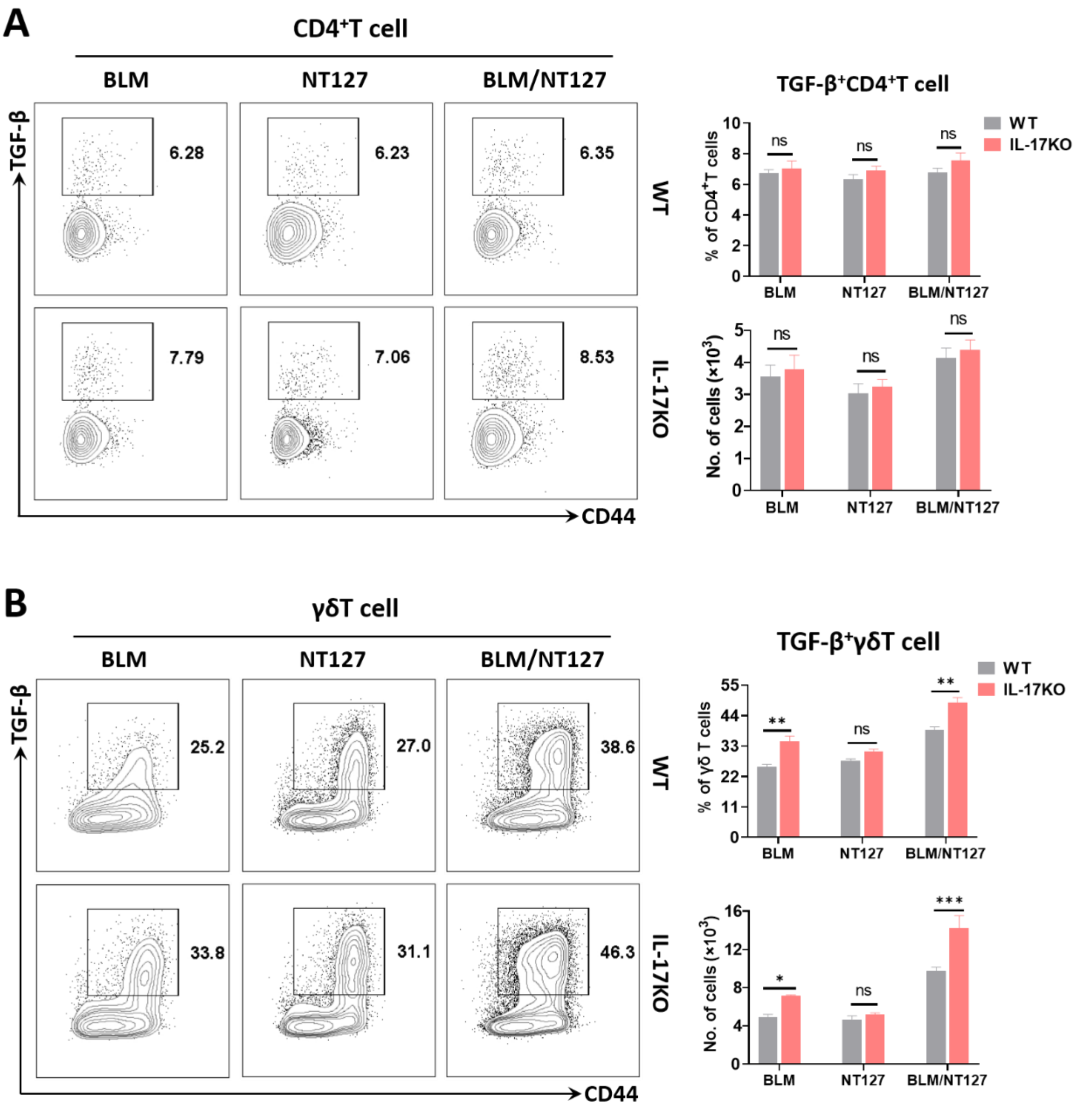

Figure S7. Impact of IL-17 on TGF-β production by pulmonary CD4$^+$ and γδ T cells in AE-IPF mice. TGF-β expression by pulmonary CD4$^+$ and γδ T cells after stimulation with PMA/Ionomcyin in WT and IL-17 KO mice were analysed by flow cytometry and calculated as the percentage and number of TGF-β$^+$CD4$^+$ **(A)** and TGF-β$^+$γδ T cells **(B)** after intranasally instilling with BLM, NT127 and BLM/NT127. Data are expressed as the mean±SEM (n=5 per group), *P < 0.05; **P < 0.01; ***P < 0.001.

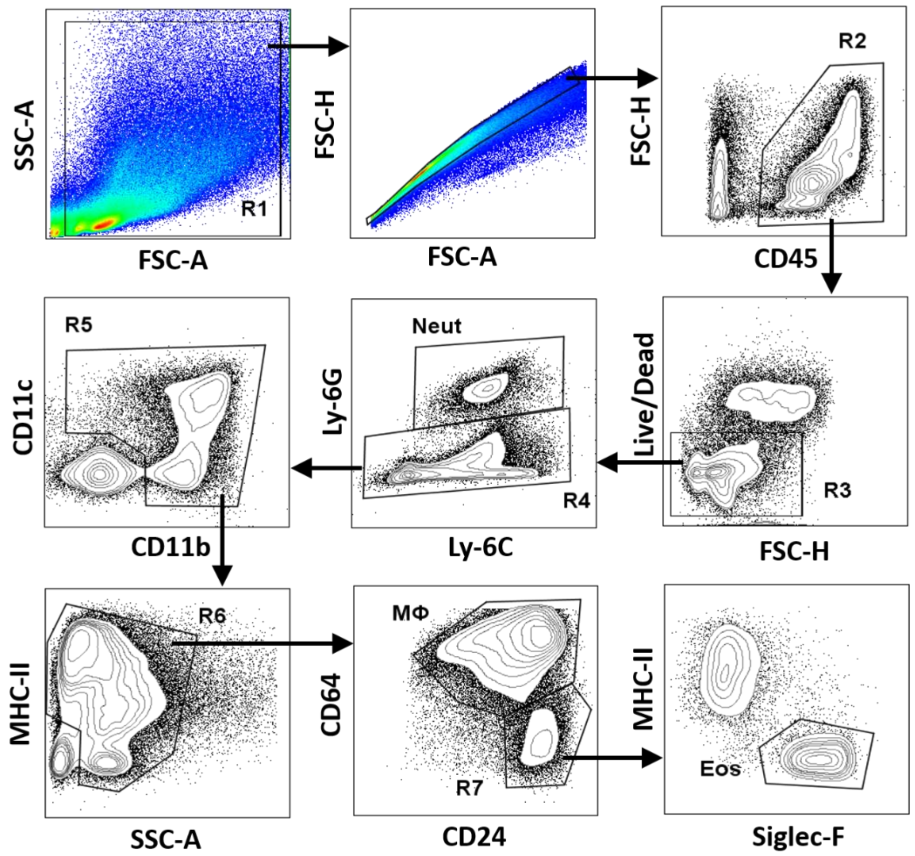

Figure S8. Gating strategy used for the identification of major inflammatory cell populations in murine lung. Gates containing multiple cell populations are numbered (R1-R7). Gates containing a single cell population are labeled with neutrophil, eosinophil and macrophage.
